# Endophytes Across the Plant Life Cycle: Potential Roles in Plant Protection, Litter Decomposition, and Nutrient Cycling

**DOI:** 10.3390/plants15162536

**Published:** 2026-08-21

**Authors:** Yumeng Sun, Xingbing He, Yonghui Lin, Zaihua He, Xiangshi Kong

**Affiliations:** 1 Hunan Provincial Key Laboratory of Ecological Conservation and Sustainable Utilization of Wulingshan Resources, School of Life Sciences, Jishou University, Jishou 416000, China; sunyumeng23@163.com (Y.S.); hezh@jsu.edu.cn (Z.H.); kongxiangshi@126.com (X.K.); 2 School of Life Sciences, Jishou University, Jishou 416000, China

**Keywords:** microbial persistence, plant–microbe interactions, saprotrophic transition, priority effects, microbial community assembly, nutrient mobilisation, ecological restoration

## Abstract

Endophytes are diverse microorganisms inhabiting plant tissues that can promote plant growth, enhance stress tolerance, or suppress pathogens. However, the ecological roles of endophytes after host senescence and death remain poorly understood. This review examines how a subset of endophytes helps maintain ecosystem function in living plants, senescent tissues, litter, and associated microbial environments. Mechanisms of endophyte-mediated plant protection in living plants include pathogen inhibition, host regulation, nutrient acquisition, and abiotic stress tolerance. We also investigate post-senescence persistence and potential roles in litter decomposition and nutrient cycling via microbial and functional legacies, priority effects, extracellular enzyme activities, and community reassembly. Vertical transmission and horizontal dispersal can move microorganisms among stages, whereas host filtering acts after arrival to determine which microorganisms establish within host tissues. However, a full same-strain life-history loop has not been established. We propose an open, non-linear ecological continuum for selected endophytes that connects endophytic colonisation, saprotrophic persistence, and potential new-host establishment. In the future, longitudinal, strain-resolved, multi-omics, and synthetic-community studies will be conducted to verify the cross-stage connections and offer a microbial framework for plant protection, litter management, and ecological restoration.

## 1. Introduction

Associations between plants and microorganisms are the fundamental biological interactions that affect plant health, stability of plant communities, and ecological functions [1,2]. Endophytes are microorganisms that inhabit internal plant tissues during all or part of their life cycle without causing obvious disease symptoms during this association [1,3]. They include diverse fungal and bacterial taxa, including Actinobacteria, and occur in the roots, stems, leaves, seeds, and other parts of plants [1,2,4,5]. Nutrient acquisition, alteration of host metabolism, stress adaptation, and pathogen inhibition are important ways by which endophytes can contribute to plant health.

Most research on endophytes has focused on their growth promotion, stress tolerance, and biocontrol of living plants. Reported mechanisms include biological nitrogen fixation, phosphate solubilization, phytohormone production, siderophore production, regulation of the antioxidant system, and biosynthesis of antimicrobial metabolites [1,2,4,5]. Certain endophytes also improve the performance of plants in metal-contaminated environments by changing the availability of metals, enhancing mineral nutrition, or reducing metal toxicity [6]. At the same time, the fate and ecological role of endophytes after host senescence, tissue death, and litter formation have received relatively little attention [7]. Due to this knowledge gap, only a host-limited view of endophyte ecology has been formed, and issues such as post-senescence persistence and endophyte-to-saprotroph transitions remain unresolved.

Decomposition of litter connects primary production with the supply of nutrients for soils and serves as a regulating factor for the cycle of carbon, nitrogen, and phosphorus [8,9]. Although decomposition is generally thought to be carried out by saprotrophic bacteria and fungi from the surrounding environment, endophytes that live inside the plant tissue can also be carried into the litter system [7]. Their previous niches may have provided early access to new substrates and allowed them to influence later-arriving decomposers through resource pre-emption, niche modification, and other priority effects [10,11].

Recently, some studies have shown that the lifestyles of endophytes and saprophytes are not always mutually exclusive or permanent. Phylogenetic evidence shows that some fungal endophytes can shift toward saprophytic nutrition after the host ages or dies [12]. Several dominant bacterial and fungal taxa that were detected during the early decomposition of *Cinnamomum camphora* leaves originated from the pre-existing endophytic community, and some became structurally important members of the saprotrophic network [13]. Similarly, some fungal endophytes that live in living European beech leaves also remained highly active in one-year-old litter [14]. Together, these studies provide evidence of partial continuity between living tissues and litter-associated stages for some taxa [7,13,14].

Together, this evidence suggests that some endophytes may have different but related functions in living plants and litter. During host life, they can promote growth and increase stress tolerance; after tissue death, some may remain to exhibit saprotrophic traits or affect the assembly of the decomposer community [1,2,4,5,6,7,12,13,14]. The above phenomena are not universal or uniformly favourable. Endophytes may accelerate, inhibit, or have no detectable effect on decomposition according to the identity of microbes, host species, litter chemistry, and other environmental factors [7]. Rapid decomposition of litter does not necessarily increase soil organic matter; other reasons may also be involved, such as microbial carbon-use efficiency, necromass production, organo-mineral interactions, etc. [15].

Therefore, this review examines endophytes across an open plant–litter–soil continuum and considers their potential recolonisation of new hosts. Persistence after host senescence may provide a potential link to subsequent recolonisation if endophyte-derived populations remain in litter or soil, disperse to new hosts, and successfully pass host filtering and tissue-entry barriers; however, this connection has not yet been directly demonstrated. The central scientific question is whether, and to what extent, endophyte-associated functions are retained, altered, or re-expressed across living plant colonisation, host senescence, litter-associated stages, and potential establishment in new hosts. It first discusses the establishment of endophytic associations in living plants and the mechanisms through which endophytes contribute to plant protection. It then focuses on their persistence after plant senescence, together with the resulting legacy and priority effects, the reassembly of microbial networks, and their roles in carbon, nitrogen, and phosphorus transformations. Finally, these processes are integrated into a broader framework encompassing microbial transmission, host filtering, and the potential recolonisation of subsequent host generations. At this time, we will distinguish between well-supported functions and conceptual cross-stage links that still require testing.

## 2. Literature Search Strategy

This narrative review was based primarily on literature searches conducted using Google Scholar and Web of Science (WoS), supplemented by reference tracking of key publications. No strict publication-date restriction was applied; the final literature set covered studies published from 1979 to 2026, with emphasis on recent peer-reviewed research. Key search terms included endophyte, senescence, litter decomposition, saprotrophic transition, nutrient cycling, and microbial transmission. Studies were selected according to their relevance to the major themes of this review.

## 3. Endophytic Colonisation in Living Plants and Mechanisms of Plant Protection

### 3.1. Establishment of Endophyte–Plant Associations

Compatible plant-endophyte associations are formed by microbial contact with the plant surface, entry into the tissue, adaptation to the endophytic niche, and persistence in host organs. Potential sources of inoculum include soil, rhizosphere microbial pools, seeds, vegetative propagules, phyllosphere communities, airborne dispersal, and plant residues [1,4,5,16,17,18]. The rhizosphere is a significant pool of bacterial endophytes; root exudates attract microorganisms to the root surface, and then, selected taxa may enter through lateral-root emergence sites, natural openings, epidermal discontinuities, or wounds to colonise cortical or vascular tissues [4,16,17,18]. Aboveground entry can occur through stomata, wounds, or epidermal microcracks, and fungal endophytes may adhere to the host, germinate, and then penetrate the host tissue by hyphal growth [1,5,18]. Seed-borne microorganisms provide another path for endophytes to reach seedlings early in plant development [1,4,18].

Entry into plant tissue does not guarantee extended retention. The endosphere is a selective environment that has been organised by tissue structure, immune recognition, antimicrobial substances, nutrient distribution, and niche limitation [1,19,20]. Stable colonisation is thus a result of interactions between microbial dispersal and entry ability, host filtering, and adaptation to the microenvironment of the tissue. Traits facilitating tissue entry may include chemotaxis and adhesion, whereas persistence after entry may depend more strongly on nutrient utilisation, immune tolerance, host adaptation, and the ability to avoid excessive defence responses [16,17,18,19]. These post-entry traits may help endophytes maintain compatibility with the host and occupy intercellular or, in some systems, intracellular niches.

Plant-endophyte associations are better viewed as dynamic states of compatibility rather than simple co-occurrence. Hosts restrict the proliferation of microbes by immune surveillance and nutrient limitation; persistent endophytes maintain a low-virulence state through evasion or counter-response to host defence systems, and utilise tissue-specific resources. Under favourable conditions, these microorganisms can provide nutrients and regulate plant hormone levels, enhance stress tolerance, suppress pathogens, etc. [1,2,4,5,19,20]. However, changes in the host’s genotype, microbial traits, environmental conditions, or community interactions can switch an association from mutualism or commensalism to conditional harm or pathogenesis [1,5,19,20]. Therefore, successful endophyte colonisation needs to be able to enter plant tissues and maintain compatibility with the host and niche.

### 3.2. Endophyte-Mediated Plant Protection

One major function during the living plant phase is endophyte-mediated plant defence. Selected endophytes can enhance the host’s performance under biotic and abiotic stress through several interconnected pathways [21,22,23]. For clarity, these pathways are divided into direct antagonism against pathogens, host-mediated protection, and context-specific expression of host-mediated mechanisms under abiotic stress. These categories are not mutually exclusive; that is to say, a single strain can have several antimicrobial substances, boost the host immune system, modify auxin signalling, enhance nutrient uptake, and increase stress tolerance at the same time [21,22,23].

#### 3.2.1. Direct Protection Against Pathogens

Direct antagonism suppresses pathogen growth, survival, virulence, or resource acquisition. The major mechanisms include antimicrobial metabolite production, secretion of pathogen-targeting hydrolytic enzymes, and competition for nutrients or ecological niches [23,24].

Some bacterial and fungal endophytes produce antibiotics, lipopeptides, phenolic compounds, terpenoids, alkaloids, volatile organic compounds, and other antimicrobial substances. These compounds inhibit hyphal growth and spore germination, damage cell membranes, and disrupt pathogen metabolism [21,22,25,26,27]. Endophytic fungi are relatively rich in bioactive natural products; at the same time, plant-associated *Bacillus*, *Pseudomonas*, and some Actinobacteria have been extensively studied for antimicrobial metabolite production and biological control [22,23,24,28].

Extracellular hydrolytic enzymes are also a type of direct antagonism. Chitinases, glucanases, proteases, and other enzymes produced by some endophytes, bacteria, and fungi can damage the cell walls or other structures of pathogens [22,23,24]. Some bacterial endophytes also release cellulases and pectinases to help enter plant tissues and move through intercellular spaces [18,22]. Since the same enzymes are also used by phytopathogens, their ecological effects are determined by the level of expression, regulatory environment, strain-specific features, and host tissue environment [18,22].

Endophytes are present in the tissue of hosts and reduce the damage caused by pathogens. Pathogens frequently multiply in the rhizosphere, rhizoplane, phyllosphere, or intercellular spaces before and during infection and require plant-derived carbon, nitrogen, and mineral nutrients. Early colonisation by endophytes or other beneficial microorganisms has reduced the availability of these resources and taken up potential infection sites [17,18,23,28,29]. Iron competition is a typical case: siderophore-producing bacteria can increase their own iron uptake and reduce the supply of iron for competing pathogens [1,4,28,29,30]. Stable niche occupation and preemptive resource utilisation in plant tissues may thus contribute to colonisation resistance [23,24].

Antimicrobial metabolites, pathogen-targeting enzymes, and competition for scarce resources all cooperate to provide direct defence for the host against pathogens. However, the actual effect varies with the strain identity, successful colonisation, host tissue, and environmental conditions [23,24].

#### 3.2.2. Indirect Protection Through Host Regulation

Host-mediated protection generally modifies the physiology or immune system of the plant to reduce the number of pathogens indirectly. Endophytes can adjust immunity and the signal-transduction pathway of plant hormones; thus, they may change root shape and reduce stress-induced accumulation of ethylene [31,32]. The above processes can increase the host’s preparedness and resilience to some extent under specific circumstances. Plant protection is used here in a wide sense to refer to the strengthening of host physiological capacity and stress tolerance, not only pathogen inhibition.

Induced systemic resistance (ISR) is a typical host-mediated mechanism. ISR does not directly inhibit pathogens but rather releases defence signals to help plants trigger an early and strong defence response during infection [31,32]. Selected endophytes can promote the defence-related signalling pathway, pathogenesis-related proteins, antioxidant systems, and defensive metabolites of the host plant to enhance its resistance to various pathogens [32,33,34].

A primed state does not generally need to have high constitutive defence activity. It will be quicker and more vigorous in the face of a pathogen attack, thus reducing the metabolic cost of continuous defence activation [31]. ISR depends on host recognition of beneficial microorganisms and their signals, and is affected by plant genotype, microbial strain, colonisation ability, nutrition, environment, etc. [31,32].

Certain endophytes and other plant-growth-promoting bacteria produce indole-3-acetic acid (IAA), which can modify the elongation of the primary root, lateral root initiation, root hair development, and the general root-system structure [35,36,37]. These changes in root architecture can improve water and nutrient acquisition. As the results are affected by IAA concentration, the host’s genotype, the microbial strain, and the environment, etc., microbially produced IAA should be viewed as a context-specific regulator of root development rather than a general growth promoter [37].

ACC deaminase-producing endophytes can degrade ACC, the immediate precursor of ethylene, and thus reduce stress-induced ethylene accumulation. This will reduce ethylene-mediated inhibition of root growth and enhance tolerance to environmental stress [35,36,38]. IAA-mediated regulation and ACC deaminase activity may interact additively, synergistically, or antagonistically depending on the microbial strain, host genotype, and environment [36,37,38], and thus should not be assumed to be the same for all endophytes.

Improved nutrient acquisition offers an extra host-mediated route. Some endophytes solubilize sparingly soluble inorganic phosphorus or mineralize organic phosphorus by producing organic acids, changing pH, altering mineral-ion chelation, and exhibiting phosphatase activity [4,35,39]. The above processes can enhance phosphorus nutrition and root development of plants; however, their actual efficacy depends on the kind of phosphorus, soil pH, microbial activity, colonisation ability, and compatibility between plants and microbes [39]. Thus, it contributes indirectly through improved host nutrition rather than direct pathogen suppression.

Therefore, ISR, phytohormone regulation, and ACC deaminase activity represent distinct processes that may operate independently or interact depending on the microbial strain, host genotype, and environment [31,32,36,37,38,39].

#### 3.2.3. Protection Against Abiotic Stress

Plants are frequently exposed to drought, salinity, heavy metal pollution, nutrient deficiency, extreme temperatures, and organic pollutants. Endophytes and other plant-associated growth-promoting microorganisms may enhance stress tolerance by modulating root development, phytohormone signalling, osmotic balance, ion homeostasis, antioxidant defence, and contaminant transformation [2,21,22,40]. Due to the different stresses imposed by these various stressors on the host, the protective effect of endophytes varies significantly among different host species and microbial strains, as well as varying degrees of stress and different environments [2,40].

Plants face water stress under drought conditions, so they close their stomata to reduce photosynthesis and are thus damaged by oxidative stress. Selected endophytes may reduce damage by modifying root-system architecture and water-use efficiency, promoting the accumulation of osmoprotectants, and enhancing antioxidant-enzyme activity [2,21,22,35,40]. IAA-mediated responses, ACC deaminase activity, and modifications in root development together promote water-use efficiency and regulate ethylene. Specific strains have increased biomass, survival, or post-stress recovery in certain host-microbe systems after inoculation [35,36,40], but these results should not be applied to all endophytes or drought conditions.

Salinity leads to ion toxicity, osmotic imbalance, and oxidative stress in excess Na^+^ [41]. Beneficial plant-associated microorganisms can reduce Na^+^ accumulation, maintain a high K^+^/Na^+^ ratio, promote compatible solute accumulation, and strengthen antioxidant defence mechanisms [2,22,38,40,42]. Endophyte-associated salt tolerance is therefore a result of coordinated regulation of root development, stress-induced ethylene, ion homeostasis, osmotic adjustment, and redox balance, rather than a single universal mechanism [2,38,40].

In metal-contaminated environments, plants face metal toxicity, nutrient imbalance, and oxidative stress. Certain endophytic strains can reduce the bioavailability of metals by surface adsorption, extracellular polymeric substances (EPS), precipitation, complexation, chelation, redox transformation, or modification of rhizosphere chemistry [6,43,44,45]. They can also promote the growth of plants, strengthen antioxidant defence systems, and alter metal uptake or translocation [6,35,40,43,44,45]. These effects are not uniform across all strains; some microorganisms can increase metal solubility to support phytoextraction, while others reduce the mobility of metals and may be more suitable for phytostabilisation. Therefore, the selected strain should be suitable for the contaminant and remediation goals [6,43,44,45].

Some plant-associated bacteria, including endophytes, can degrade, transform, or co-metabolise certain compounds of organic pollutants to reduce pollutant concentrations or phytotoxicity [43,44]. Plant-microbe systems can increase the remediation effect through a combination of plant uptake and microbial modification of rhizosphere or internal tissue environments. These functions are related to the chemistry of contaminants, the metabolic abilities of microbes and hosts, and colonisation efficiency, so they cannot be generalised to all endophytes [44].

Karst ecosystems show how several constraints can act simultaneously. Shallow soil, irregular water supply, low phosphorus availability, high calcium concentration, and other nutritional deficiencies in plants have been reported [46]. Some endophytes can increase the mobilisation of sparingly soluble phosphorus, improve phosphorus utilisation efficiency, and promote root growth [4,35,39]. Plants with different calcium-adaptation strategies have different foliar endophyte bacterial communities, and these communities are related to the ecological strategies of the hosts [46]. The above patterns suggest that functionally appropriate endophytes may improve plant performance under nutrient-limited karst conditions; however, their specific reasons for long-term adaptation have not been clarified [2,35,39,46].

In all these stress situations, endophyte-induced protection generally works by regulating host physiology, nutrition, and stress-responsive plant-environment interactions. Abiotic-stress protection is therefore not independent of host-mediated protection; rather, it is the result of overlapping regulatory mechanisms under specific adverse conditions [40].

**Figure 1:**
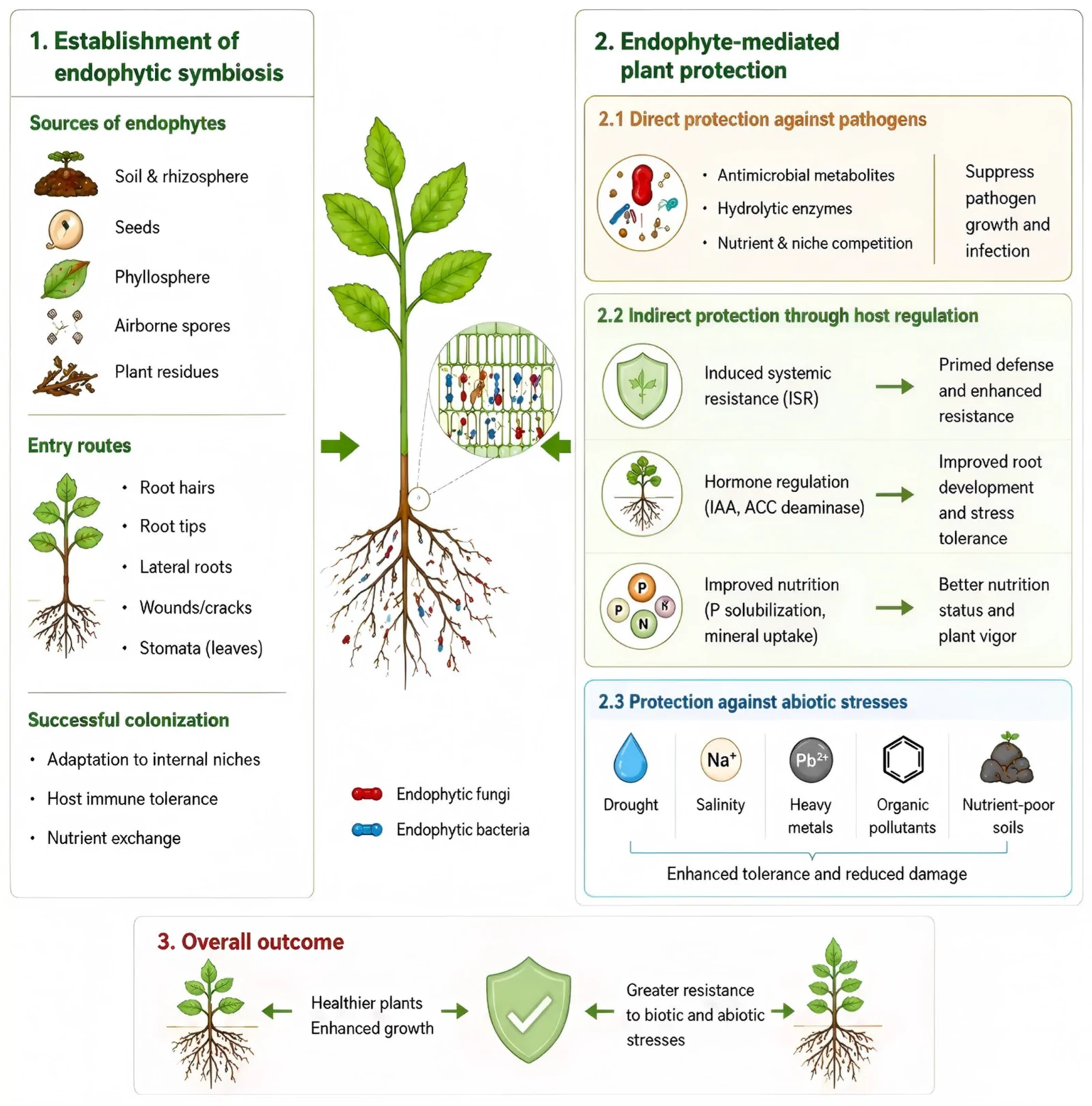
Mechanistic framework of plant–endophyte associations and endophyte-mediated plant protection. Selected endophytes may enhance various functions of plants through tissue colonisation, such as direct pathogen inhibition, host immune system regulation, improved nutrient acquisition, and stress tolerance.

Endophytes can protect the host during the living plant stage by directly antagonising pathogens, modifying the host, or inducing stress-specific responses in the host. The above pathways can work together in the same strain–host system and should not be considered independently Figure 1. Host senescence alters tissue conditions, nutrient availability, and microbial niches, which may allow some established endophytes to persist and shift toward litter-associated or saprotrophic functions, as discussed in the following section.

## 4. Litter Stage: Endophyte-Associated Regulation of Decomposition and Nutrient Mobilisation

### 4.1. Functional Legacies and Priority Effects of Endophytic Pre-Colonisation

When the host tissue ages and turns into litter, some resident endophytes may also enter the decomposition system instead of disappearing immediately. Evidence for post-senescence persistence and functional transition comes from various phylogenetic, longitudinal, community-tracking, and controlled-inoculation studies [12,13,14,47,48]. Here, taxonomic continuity refers to the detection of the same taxa across stages, strain continuity to the persistence of the same strain, and functional continuity to the retention or re-expression of comparable functions; evidence for one does not necessarily demonstrate the others. Therefore, the death of the host does not necessarily mean that ecological life for all endophytes has ceased; for some groups, there will be changes in nutrient supply and altered ecological niches and functions. Phylogenetic analysis indicates that some fungal endophytes can shift toward a saprotrophic mode of life after host senescence [12], and the proposed endophyte-to-saprotroph continuum describes a possible shift from living tissues to dead plant residues via senescent tissues [13].

Endophyte-associated carryover effects can be divided into the following three types conceptually. Microbial legacy refers to the process in which organisms already inhabiting living tissues are transported to the litter environment and influence the initial construction of saprotrophic communities. Their previous niches of residence may have provided earlier access to substrates than what soil- or air-borne immigrants have been able to obtain, thus forming the basis for priority effects [7,14,49]. A functional legacy refers to the persistence of enzyme production, nutrient-mobilisation ability, secondary-metabolite synthesis, or other resource-utilising characteristics after host death [7,47,49,50]. Substrate legacy refers to alterations in the elemental composition, secondary metabolites, and other chemical features of plant tissues by endophytes during their lifetime, which consequently modify the quality of subsequent litter [5,7,47,49]. These legacy mechanisms are not mutually exclusive and may interact to shape subsequent community assembly and decomposition. Therefore, legacy effects refer to persistent changes in community composition, functional traits, or substrate properties resulting from prior microbial occupancy, including effects that persist after host senescence or tissue death.

Controlled experiments provide direct evidence of post-senescence effects. In an *Arabidopsis thaliana* litter system inoculated with the endophyte bacterium *Bacillus cereus*, the mass loss of litter increased during months 2–4, early alkaline phosphatase activity was higher, final soil available phosphorus increased, and soil bacterial diversity and composition changed [47]. The above results indicate that, under the conditions of this experiment, an endophyte affected phosphorus mobilisation and soil microbial community structure. These findings should not be generalised to all microbial taxa or litter types; rather, the degree and direction of the effect are likely to differ according to microbial identity, substrate characteristics, community structure, and environmental conditions [39,47].

However, pre-colonisation alone does not demonstrate a true priority effect, because early community assembly may also reflect differences in initial abundance or substrate modification. Controlled arrival-order experiments will therefore be particularly useful for separating true priority effects from these alternative mechanisms. Because endophytes live in plant tissues before abscission, they may find new substrates first, outcompete external colonisers, and act as pioneer decomposers [7,12,14,48,50]. Early litter has relatively labile compounds, such as soluble sugars, amino acids, low-molecular-weight organic acids, and soluble phosphorus fractions [8,9]. Endophyte-derived microorganisms may use these resources to alter the initial transformation of carbon, nitrogen, and phosphorus via cellulases, hemicellulases, β-glucosidases, phosphatases, organic acids, and other metabolites [7,47,50,51].

The results of the initial colonisation are not confined to the early settlers’ bodies. Resource pre-emption, niche modification, and alteration of the local physicochemical environment can all hinder or promote the development of later-arriving saprophytic bacteria and fungi by early endophyte-derived populations [10,11,48,49,50,52]. Research on the decomposition of wood and *Quercus acutissima* litter has shown that endophyte communities or individual strains can change subsequent microbial assembly, extracellular enzyme activity, litter mass loss, and the abundance of functional groups [48,49,50]. Therefore, an analytically convenient sequence is priority colonisation—niche modification—community reassembly, although the strength and direction of each step vary by system.

### 4.2. Microbial Community Succession and Network Reconfiguration During Decomposition

After the first colonisation period, endophyte-derived populations may still affect litter decomposition through community succession and network restructuring. Litter communities are continuously supplied with inputs from the phyllosphere, endosphere, soil, and air, and show significant taxonomic turnover and functional restructuring [51,52]. In the decomposition of *Cinnamomum camphora* leaves, bacterial and fungal taxa that were endophytes played a structural role in the early saprotrophic network, and network properties and decomposition-related functions were associated with endophyte-derived dominant or candidate keystone taxa [13]. Based on the above observations, some endophyte-derived populations may affect the start of decomposition as well as the structure and function of later litter communities.

Co-occurrence networks can find highly connected taxa, module hubs, and taxa that connect modules, but network position alone does not show ecological causality. Microbial diversity and network complexity are related to ecosystem multifunctionality, and experimental isolation has shown that some network-inferred candidate keystone taxa have strong litter-degrading capabilities and extracellular enzyme activities [53,54]. Endophyte-derived candidate keystone taxa need to be verified by isolation, reinoculation, removal, or synthetic-community experiments before they are considered drivers of decomposition. The potential consequences of these may include changes in community cooperation, network structure, and enzyme-function modules [13,50,53,54].

Therefore, the post-senescence effect of endophytes can be classified into the following three non-mutually exclusive analytical dimensions: functional and substrate legacies, priority effects during early colonisation, and subsequent community succession or network reconfiguration. These attributes may overlap and are not necessarily in a fixed order in time. Together, they provide the community-level context for the effect of endophyte-derived microorganisms on carbon, nitrogen, and phosphorus cycling.

### 4.3. Nutrient Transformation and Mobilisation During Litter Decomposition

Saprotrophic endophytes can also release extracellular enzymes to decompose substrates and obtain nutrients in other ways during decomposition. The capacity is not the same for all endophytes, and it is not always present after decomposition; rather, it is specific to different times and environments and depends on microbial characteristics, substrate quality, community structure, and so on [7,12,49,50]. Potential functions include the degradation of cellulose and hemicellulose and the conversion of organic nitrogen and phosphorus pools [7,12,47,50,54]. Therefore, the endophytic origin should not be taken as evidence of continuous saprophytism.

Cellulose, hemicellulose, and lignin are the main components of litter for carbon cycling that affect the decomposition rate. Endophyte-derived microorganisms have built extracellular enzyme systems to break down easily degradable carbon sources early in decomposition and increase substrate availability by altering plant cell walls [12,50,51]. Lignin degradation requires special oxidative enzymes, and therefore is more closely associated with fungal taxa that possess such enzymatic capabilities [55]. Therefore, the contribution of endophyte-derived populations to carbon release varies according to enzymatic repertoire, decomposition stage, litter chemistry, and interaction with later-arriving saprotrophs [7,12,48,50,51]. Moisture, acid deposition, nitrogen input, and understory management also change microbial activity, extracellular enzyme activity, and substrate availability, so the effect of endophytes needs to be considered in the context of the environment [56,57,58,59].

Organic nitrogen in litter can be transformed through protein decomposition and microbial uptake. Microbial uptake may lead to nitrogen immobilisation in microbial biomass, whereas nitrogen mineralisation can result in the release of inorganic N. Endophytic bacteria in the living plant stage can help the host obtain nitrogen, and some taxa have nitrogen-fixing or other nitrogen-transforming abilities [60,61]. There is no direct evidence to confirm that these functions are still present in the endophyte-derived population after it enters the litter. Taxa that retain proteolytic, ammonifying, or other characteristics may influence local nitrogen transformations; however, the actual effect depends on factors such as litter C:N ratio, moisture, temperature, pH, and external immigration and competition [9,52,62]. Therefore, post-senescence regulation of litter nitrogen mineralisation by endophyte-derived microorganisms should be considered a testable hypothesis rather than an established general function [7,60,63].

Phosphorus cycling can be carried out by endophyte-derived microorganisms to increase phosphate availability through phosphate solubilization and phosphatase activity. At the living-host stage, some endophytes sparingly mobilise insoluble inorganic phosphorus or mineralize organic phosphorus by means of organic acids, phosphatases, and other metabolites [22,39]. These abilities may be kept or shown in another way after entering litter. A defined *Bacillus cereus*–litter system was used, and inoculation increased alkaline phosphatase activity and soil-available phosphorus [47], thus directly demonstrating localization of phosphorus mobilisation. However, the generalisations and directions of this effect are also influenced by microbial traits, phosphorus composition in the substrate, interactions with other decomposers, and environmental conditions [7,8,9,39,47].

Endophyte-associated alterations in decomposition may also affect the production of soil organic matter indirectly. Soluble compounds and mineral nutrients released by litter are absorbed and changed by soil microorganisms, and some of these substances may enter a relatively stable pool as microbial products, necromass, and organo-mineral associations [15]. Therefore, an increase in decomposition does not necessarily lead to more net carbon loss or an increase in soil organic matter. The actual consequences are related to substrate decomposition, microorganism carbon utilisation efficiency, necromass generation, and mineralisation [15].

**Figure 2:**
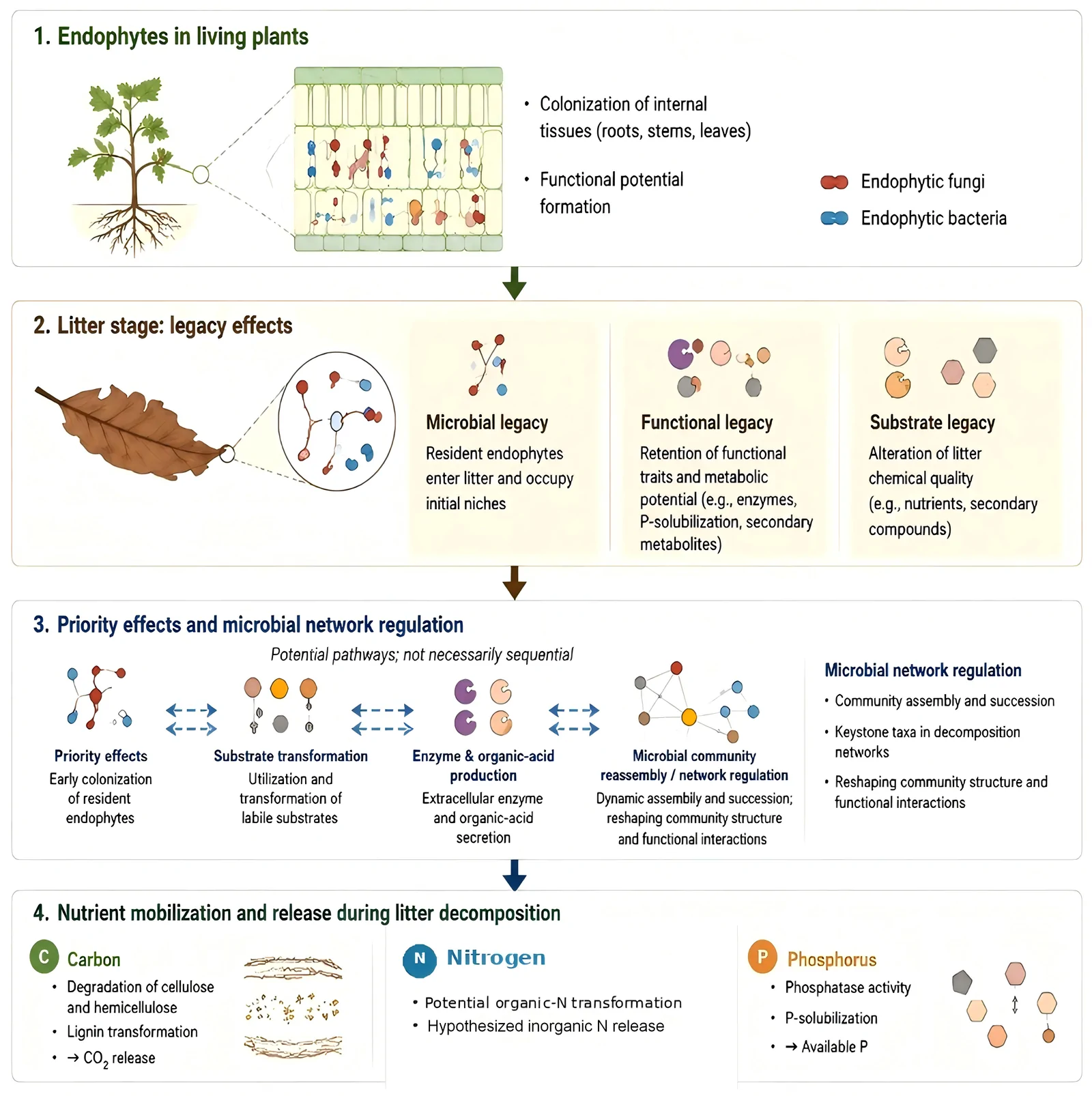
Conceptual model of how endophyte-associated legacy effects and priority effects may influence litter decomposition, microbial network reassembly, and litter carbon transformation and phosphorus mobilisation through multiple, non-sequential pathways. Nitrogen transformation is offered as a possible path because no direct post-senescence evidence has been found.

In general, endophyte-derived microorganisms can have different effects at different stages of litter decomposition and local C, N, and P transformations through several interacting pathways: legacy effects, priority colonisation, extracellular enzyme and organic acid production, substrate modification, nutrient release, and community restructuring Figure 2. These paths may occur in sequence or simultaneously, and some may not exist in a particular system. At present, the strongest evidence is for the early community effect and localised phosphorus mobilisation; continuous changes in nitrogen cycling, soil carbon storage, and cross-generational host transmission have not yet been observed.

## 5. Ecological Continuum of Endophytes: From Colonisation to Saprotrophic Persistence and Potential Recolonisation

The ecological conditions of the endophyte can be altered during changes in the host, ageing of tissues, environmental disruptions, dispersion, and recolonisation [1,5,64]. An endophyte is a microorganism that lives in the tissues of a living plant for all or part of its life cycle without causing obvious disease at the time of association; this association is not necessarily permanent [1,3,5]. According to the developmental stage of the host, tissue niche, immune status, microbial traits, and community context, plant-endophyte associations may vary among mutualism, commensalism, latent pathogenicity, and conditional pathogenicity [1,5,61,62,64,65,66,67,68].

Endophyte life histories are therefore better represented as an open continuum than as a closed cycle. Endophytes may be mutualistic, commensal, or latent colonisers in the living stage. A fraction may be subject to senescence or abscission and enter the litter or soil pool for early decomposition or microbial succession [5,12,13,14]. Other populations may be vertically transmitted through seeds or vegetative propagules, and soil, litter, air, insects, and surrounding plants provide sources for horizontal acquisition [65,67,69,70,71,72]. Establishment in a new host is still subject to arrival, host filtering, tissue entry, adaptation, and competition [17,18,19,65,67].

### 5.1. Stage-Dependent Ecological Transitions

With continuous changes in tissue structure, carbon availability, secondary metabolites, immune filtering, and physicochemical conditions during plant development, selection occurs at different stages for incoming and established microorganisms [1,61,64]. Endophytic communities are generally more constrained by host filtering than rhizosphere or phyllosphere communities, and successful colonisers need to adapt to host nutrient supply, immune recognition, and tissue-specific niches [64,67,68]. Therefore, the life history of an endophyte is a series of selections and functional adaptations that occur dynamically after entry and establishment, rather than a simple process of entry and establishment.

Seed-associated microorganisms can provide an initial inoculum for the newly established plants. Longitudinal and experimental transmission studies indicate that some seed-associated microorganisms can reach roots, shoots, the phyllosphere, or other plant-associated compartments after germination and contribute to subsequent microbiome assembly [69,70,71,72,73]. However, the early endophyte community is not a direct copy of the parental microbiome; rather, it results from the combined effects of seed transmission, horizontal acquisition, root exudates, immune filtering, and competition. Seed-associated endophytes are therefore best regarded as an initial inoculum source rather than a stable endpoint community. Importantly, direct strain-resolved evidence tracking the same endophytic taxa or strains continuously from seed-associated stages through senescence and into litter remains limited, and complete cross-stage continuity has yet to be demonstrated.

Roots, stems, leaves, and other organs provide relatively stable but tissue-specific endophyte niches during vegetative growth. Hosts select microbial taxa based on exudates, nutrient gradients, cell-wall structure, and immune regulation, and endophytes alter plant phenotypes through metabolic interactions, nutrient modification, growth regulation, and stress response [21,60,64]. Community composition and function also vary with the stage of development, nutritional status, and seasons [74,75].

Microorganisms may colonise reproductive tissues, including flowers, pollen, ovules, and fruits, potentially providing opportunities for seed-mediated vertical transmission. However, colonisation of reproductive tissues does not by itself demonstrate stable entry into mature seeds or persistence in the next generation [69,70,71,72,76].

During senescence, changes in host regulation, tissue chemistry, and cell-wall properties may create new ecological opportunities for resident microorganisms. Taxa with suitable substrate utilisation, extracellular-enzyme, and competitive traits may persist as early saprotrophs or influence the initial decomposer community [12,13,14,47,48,49,50,51,52,77]. However, such transitions are neither universal nor inevitable. These changes may act jointly: reduced host filtering alters the selective constraints on resident microorganisms, newly available substrates favour taxa with suitable resource-utilisation traits, and increasing competition from external saprotrophs further determines which endophyte-derived populations persist and how their functions change. Host senescence thus creates a new selective environment in which some endophyte-derived populations are retained, others are lost, and externally arriving saprotrophs become increasingly prevalent. These potential role transitions are summarised in Table 1.

### 5.2. Transmission and Potential Recolonisation of New Hosts

One potential route is dispersal among hosts and environmental compartments for potential recolonisation. Soil, litter, rain splash, insects, air, and neighbouring plants can provide horizontally acquired inocula; however, the relative contributions of these sources vary according to host species, tissue type, and environmental conditions [13,51,52,65,67]. Endophyte-derived microorganisms in the litter may remain for a short time and affect local microbial succession or nutrient cycling [13,47,49,50,52]. It has not been determined whether these populations provide inoculum for new hosts at present.

Arrival at the plant surface does not lead to an endophyte association. Microorganisms need to attach to tissues, enter them, cope with or change host defence mechanisms, adapt to the internal environment, and compete with other communities [16,17,19,67]. Therefore, transition from a litter- or soil-associated saprotrophic state to an endophytic association in a new host should not be assumed to occur automatically; it requires successful dispersal, host recognition, tissue entry, adaptation to the internal environment, and competition with resident microbial communities.

Vertical transmission is a parallel path of intergenerational inheritance that mainly uses seeds or vegetative propagation. Microorganisms may also be present in the pollen or other reproductive organs, but pollen-to-seed transmission has not been fully elucidated [76]. Pollinators may facilitate horizontal microbial dispersal among flowers and host plants. Seed-borne endophytes can affect germination, early disease resistance, or stress adaptation [71,72,73,78,79], but vertical transmission is not stable inheritance; maternal environment, flowering, fruit development, seed maturation, dormancy, and storage all influence the efficiency of transmission and seed-associated community composition [69,70,72,78,79].

Community establishment in a new plant can be shown as arrival-filtering-colonisation-stabilisation. First, microorganisms may reach host plants via seeds, soil, litter, air, and biological vectors. Arrival order can then generate a priority effect through spatial pre-emption, resource use, niche modification, or host-mediated responses [10,11]. Entry into the internal tissues is regulated by root exudates, tissue nutrients, immune recognition, developmental stage, and host genotype [61,67,68,83]. Finally, microorganisms need to form relatively stable associations with host tissues and the resident community in plants to remain in the plant-associated microbiome [61,66].

**Figure 3:**
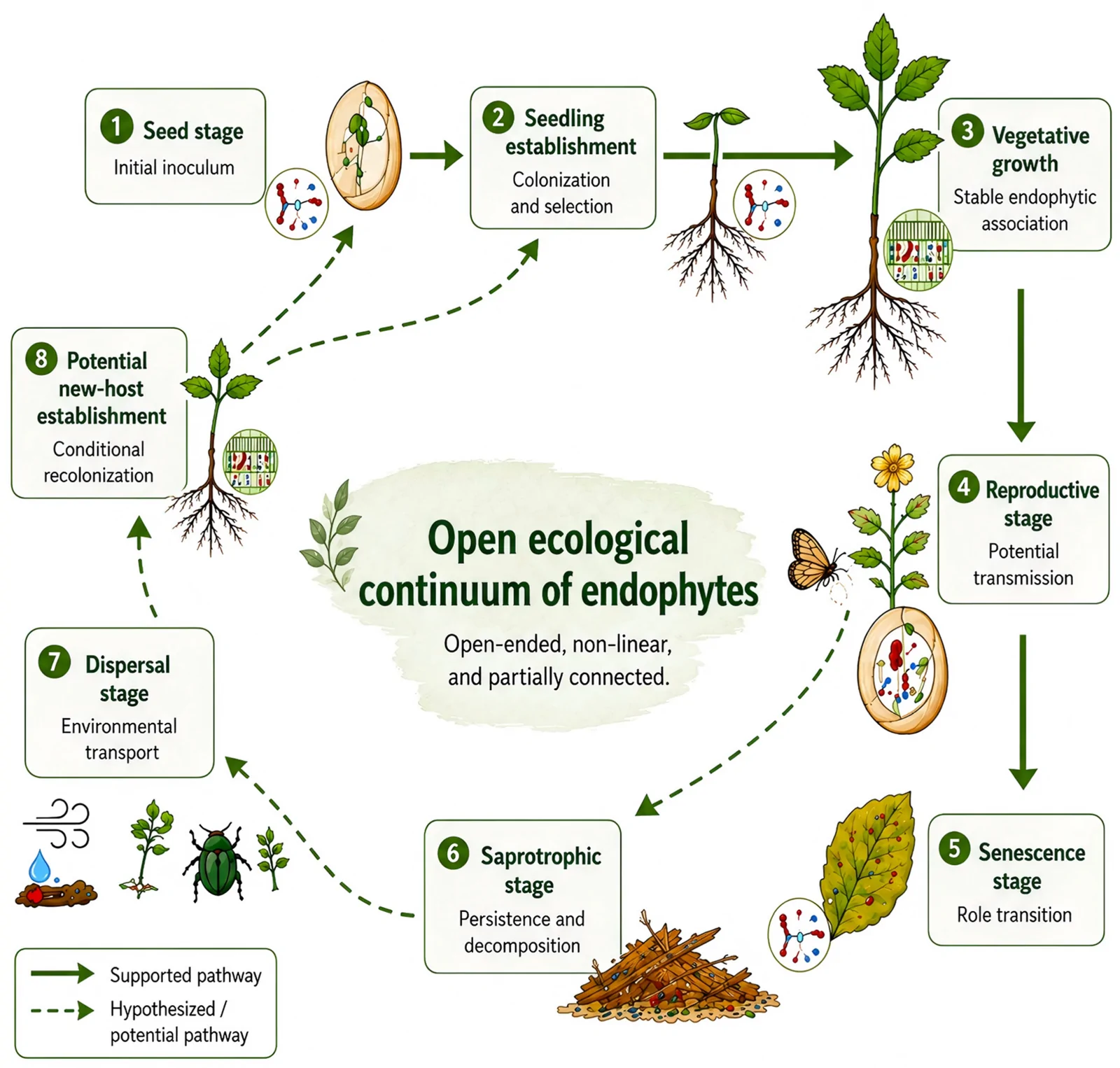
Conceptual model of the open ecological continuum of endophytes. Vertical transmission, horizontal acquisition, potential saprotrophic persistence, environmental dispersal, renewed host filtering, and possible recolonisation are loosely connected parts; the figure does not suggest that a complete same-strain loop occurs everywhere.

Therefore, the proposed ecological continuum is open-ended and non-linear. Vertical transmission, horizontal dispersal, saprotrophic persistence, and renewed host filtering are partially independent paths that may connect plant-associated and environmental microbial stages. If a compatible association is formed in a new host, plant-protection functions may be displayed again; however, neither taxonomic continuity nor functional equivalence should be presumed Figure 3.

Under certain combinations of host identity, microbial taxon, and environmental conditions, some endophytes may move among seed-associated, vegetative, reproductive, senescent, litter-associated, environmental, and newly colonised host stages. Therefore, we employ the concept of “endophytic colonisation in living plants—saprotrophic persistence—potential recolonisation of new hosts” as a framework to integrate partially connected evidence. This framework should not be interpreted as evidence that a complete same-strain loop occurs in all natural systems.

### 5.3. Environmental Regulation of Life-Cycle Transitions

Environmental stress can regulate endophyte colonisation, functional expression, and community structure during the living plant stage. Drought, salinity, extreme temperatures, nutrient deficiencies, heavy metal stress, and other factors affect host water relations, phytohormone signalling, redox balance, root exudates, and tissue microenvironments, thereby altering endophyte distribution and community structure [84,85]. Whether these environmental effects also determine which taxa persist during senescence or enter the saprotrophic stage remains less directly demonstrated and should therefore be regarded as a plausible cross-stage inference requiring further validation.

Drought is a typical case. Extended water limitation modifies root exudation, alters osmotic conditions, changes tissue water content and defence-related signals, and thus directs selection for rhizosphere and endophytic communities. Prolonged drought in rice led to persistent changes in the composition of the root-associated microbiome, with root endophytic communities showing greater sensitivity than rhizosphere communities and certain bacterial taxa exhibiting long-term enrichment [86]. Such patterns are consistent with microbial contributions to host adaptation, but cultivation-based assays, synthetic communities, and functional experiments are needed to establish causality [2,84,85,86].

Stress may also alter fungal vegetative growth, latency, sporulation, propagule production, dispersal, and saprotrophic transition [5,12,14]. Direct evidence is still relatively rare, and the response may be taxon-specific. Therefore, the most justifiable interpretation is that environmental change alters host physiology and microbial niches, creating a trade-off among low-abundance colonisation, stress-tolerant persistence, reproduction, dispersal, and saprotrophic activity. Environmental conditions may determine which endophyte-derived populations persist at different stages, but they do not prescribe a single life-history strategy.

## 6. Conclusions and Perspectives

### 6.1. Major Conclusions

Plant endophytes have long been studied in living tissues to explore their effects on plant growth, stress tolerance, and disease resistance. Based on the accumulated evidence, it is proposed that the ecological relevance of some endophytes may extend to senescent tissues and litter-associated microbial succession. Therefore, endophytes should be viewed as plant-associated microorganisms that may also influence various links in the plant-tissue-litter-soil-microbe-nutrient cycle and other processes.

During the living plant stage, endophytes can contribute to plant growth, stress tolerance, and disease resistance through multiple mechanisms. However, the expression and ecological consequences of these functions depend on microbial identity, host identity, tissue niche, developmental stage, and environmental conditions. Plant–endophyte relationships may therefore shift among mutualistic, commensal, latent pathogenic, and conditionally deleterious states.

After senescence or tissue death, a subset of endophytes or endophyte-derived populations may persist in litter and influence early microbial succession, decomposition, and localised nutrient transformations. Current evidence is stronger for effects on early community assembly, litter carbon transformation, and localised phosphorus mobilisation, whereas sustained regulation of litter nitrogen mineralisation, soil carbon sequestration, and ecosystem-scale nutrient cycling remains uncertain.

Vertical transmission, horizontal acquisition, environmental dispersal, and renewed host filtering may link living-tissue colonisation, post-senescence persistence, and the formation of plant-associated communities in subsequent generations. Therefore, we put forward an open and non-linear continuum connecting “endophytic colonisation in living plants—saprotrophic persistence—potential recolonisation of new hosts.” This framework integrates related but incomplete cross-stage processes. A complete same-strain life-history loop should therefore be regarded as a testable hypothesis rather than an established general pattern.

This framework may guide the development of microbial strategies for plant defence, sustainable agriculture, environmental remediation, and ecological restoration. However, the beneficial functions in life cannot be assumed to continue after the host dies, and post-senescence effects may be beneficial, neutral, or detrimental. Accordingly, potential applications should be based on stage-specific functional verification rather than taxonomic identity or in vitro performance alone.

### 6.2. Future Research Directions

Although considerable progress has been achieved, the persistence, functional transitions, dispersal, and establishment of endophytes across living plants, litter, soil, and new hosts remain insufficiently resolved, particularly whether the same taxa or strains persist continuously from seed-associated stages through senescence and into litter. Future research should prioritise long-term observations, strain-resolved tracking, multi-omics analysis, controlled ecological experiments, and application-oriented validation. A major objective is to distinguish between taxonomic stability and functional stability, and to find causes that are reproducible and ecologically relevant.

Amplicon sequencing and metagenomics are useful tools for investigating community composition and functional potential [87], but they cannot, by themselves, reveal which taxa actively participate in plant defence, litter decomposition, and nutrient cycling. Future studies should integrate strain-resolved metagenomics with metatranscriptomics, metabolomics, proteomics, stable-isotope probing, and spatially resolved sampling. These multi-omics approaches should be used to link host gene expression, microbial traits, and plant phenotypes [88]. Comparable designs of senescence and decomposition can be used to determine whether certain strains are preserved, whether their functions have been retained or re-expressed, and whether similar functions are carried out by newly formed taxa.

As most of the present evidence is correlational, controlled experiments are needed to determine cause and effect. Gnotobiotic plants, synthetic microbial communities, microbial removal and reinoculation, stable-isotope tracing, strain-specific markers, and long-term litter incubations can be employed to track microorganisms from living tissues through senescence and litter formation. Synthetic communities can be used to test causal relationships among microbial composition, host responses, and ecological functions [89]. Such experiments should determine whether the same strain persists across stages, whether its activity changes after host death, and whether it can subsequently establish an endophytic association in a new host.

Endophytes participate in interaction networks among hosts, pathogens, decomposers, and other microorganisms in the environment. Co-occurrence networks and ecological models can help identify candidate taxa and interaction modules [90,91], but statistical centrality does not indicate ecological causality. Network-inferred candidate keystone taxa should therefore be tested by isolation, reinoculation, removal, synthetic-community reconstruction, or functional-gene validation before being identified as drivers of plant health, community assembly, or ecosystem processes.

A key long-term objective is to develop a microbial inoculation system that addresses both living plant functions and post-senescence behaviour. At present, the main applications are growth promotion, disease suppression, stress tolerance, and remediation [66,92,93,94], and research on lifestyle transition and litter has indicated that some endophyte-derived microorganisms may persist after senescence or affect litter decomposition [12,13,14,47,49,50]. These lines of evidence support an integrated experimental framework, but a single inoculant that can simultaneously promote plant protection, litter decomposition, and ecological restoration has not yet been found.

Future inoculant studies need to track the strains after host death and determine whether they are still detectable and active, whether relevant functions have been retained or re-expressed, and whether there are beneficial, neutral, or detrimental effects on decomposition and nutrient mobilisation. Synthetic communities can combine strains with different traits for controlled experiments on interaction and functional stability [89]. All proposed functions of multifunctionality need to be verified independently in living plant colonisation, post-senescence persistence, litter decomposition, and potential recolonisation.

Potential applications include environmentally friendly agriculture, disease control for plants, remediation of contaminated areas, and possibly in the future, management and regulation of nutrients in waste [93,94]. Progress in field application will need to consider strain persistence, biosafety, host specificity, environmental stability, non-target effects, and consistency under real-world conditions. Litter-management applications should be viewed as research targets and are not yet mature technologies.

Current evidence remains limited in taxonomic and geographic coverage. Most of the direct evidence is obtained from a small number of host species, organs, and forest-litter systems, and only a few studies have been conducted in subtropical and karst environments. Therefore, the generality of the proposed patterns across plant lineages, agricultural systems, climates, organs, and decomposition environments is unknown and needs to be verified by coordinated multi-site and cross-host experiments.

Future progress will therefore depend on experimental systems that track both strain persistence and function across ecological stages. Rather than assuming functional stability from taxonomic persistence, future studies should independently verify colonisation, post-senescence persistence, ecological effects, and recolonisation capacity under controlled and field conditions.

## Figures and Tables

**Table 1:** Conceptual Summary of Potential Ecological Role Transitions for Endophytes in Plant Development and Litter Stages.

Stage	Potential Ecological Role	Major Drivers	Key Mechanisms	Potential Ecological Functions	References
Seed stage	Seed-borne endophytes; an initial inoculum reservoir for the plant microbiome	Maternal environment, seed development, and vertical transmission	Retention of microorganisms within seeds, migration during germination, and early host filtering	Establishment of the early seedling microbiome and modulation of early plant development and stress tolerance	[69,70,71,72,73,78,79,80]
Seedling establishment	Initial colonisers	Root exudates, soil microbial reservoirs, and host immune status	Rhizosphere recruitment, entry into plant tissues, immune tolerance, and niche adaptation	Establishment of early endophytic communities and potential enhancement of seedling establishment and stress tolerance	[16,17,18,19,61,64,67,81]
Vegetative growth	Persistent endophytic colonisers	Host genotype, tissue type, nutritional status, and environmental variation	Host filtering, metabolic interactions, phytohormone regulation, and nutrient exchange	Promotion of plant growth and enhancement of disease resistance and abiotic stress tolerance	[1,21,61,62,64,67,68]
Reproductive stage	Endophytes colonising reproductive tissues and potentially transmitted across generations	Flower, fruit, and seed development and pollination processes	Seed-mediated vertical transmission and putative pollen-associated microbial transfer	Potential contribution to intergenerational continuity in plant–microbe associations	[69,70,71,72,76,78]
Senescence stage	A subset of transitional populations with the potential to shift toward saprotrophic lifestyles	Host death, changes in tissue chemical composition, and resource release	Potential transition toward saprotrophic activity and expression of saprotrophic capacities	Potential retention or restructuring of selected functions during the transition from living tissues to litter-associated processes	[5,12,13,14,48,77]
Early litter decomposition	Potential pioneer decomposers and community modifiers	Endophytic origin, litter chemistry, decomposition stage, and competition from externally arriving microorganisms	Functional legacy, priority effects, changes in extracellular enzyme activity, and resource pre-emption	Decomposition of plant-derived carbon compounds and potential nutrient mobilisation, with comparatively strong evidence for localised phosphorus mobilisation	[7,12,14,47,48,49,50,51]
Late decomposition	Dominant saprotrophic taxa and network-inferred putative keystone candidates	Resource shifts, microbial immigration and competition, and environmental filtering	Community succession, network reconfiguration, and formation of functional modules	Potential regulation of community succession and decomposition-related nutrient processes	[13,49,50,52,53,54]
Transmission and potential recolonisation	Potential dispersal and recolonisation candidates	Vertical transmission, horizontal acquisition, environmental microbial sources, and renewed host filtering	Arrival–filtering–colonisation–stabilisation	Contribution to renewed plant-associated community assembly and potential establishment of endophytic associations	[10,11,16,17,18,19,65,67,68,69,70,71,72,73,78,82]

## Data Availability

No new data were created or analysed in this study. Data sharing is not applicable to this article.

## Abbreviations

The following abbreviations are used in this manuscript:

ISR	induced systemic resistance
IAA	indole-3-acetic acid
ACC	1-aminocyclopropane-1-carboxylate
EPS	extracellular polymeric substances
